# ST-elevation myocardial infarction following systemic inflammatory response syndrome

**DOI:** 10.5830/CVJA-2014-071

**Published:** 2015

**Authors:** Ying Tan, Yan Tu, Di Tian, Chen Li, Jian-Kai Zhong, Zhi-Gang Guo, Ying Tan

**Affiliations:** Division of Cardiology, Nanfang Hospital, Southern Medical University, Guangzhou, Guangdong, China; Division of Cardiology, Nanfang Hospital, Southern Medical University, Guangzhou, Guangdong, China; Division of Cardiology, Nanfang Hospital, Southern Medical University, Guangzhou, Guangdong, China; Division of Cardiology, Nanfang Hospital, Southern Medical University, Guangzhou, Guangdong, China; Division of Cardiology, Nanfang Hospital, Southern Medical University, Guangzhou, Guangdong, China; Division of Cardiology, Nanfang Hospital, Southern Medical University, Guangzhou, Guangdong, China; Division of Cardiology, The First People’s Hospital of Shunde, Guangdong, China

**Keywords:** systemic inflammatory response syndrome, ST-elevation myocardial infarction

## Abstract

Systemic inflammatory response syndrome (SIRS) complicated with ST-elevation myocardial infarction has rarely been reported, and the precise mechanisms of myocardial injury remain unclear. Here, we present a case involving a 45-year-old man who developed SIRS secondary to diabetesinduced infection, and who ultimately developed ST-elevation myocardial infarction with acute heart failure, fulminant diabetes, acute liver dysfunction, acute kidney dysfunction and rhabdomyolysis. The patient eventually recovered due to early detection, correct diagnosis and powerful treatment. Clinicians should be aware of this new type of myocardial infarction, which is induced by inflammatory injury, but is not due to a primary coronary event such as plaque erosion and/or rupture, fissuring or dissection.

## Abstract

Systemic inflammatory response syndrome (SIRS) results from a variety of severe clinical insults, such as infection, pancreatitis, trauma, surgery or other critical illness, and its diagnosis^1^ includes the presence of at least two of the following: (1) heart rate > 90 beats/min; (2) respiratory rate > 20 breaths/ min or carbon dioxide pressure (PaCO_2_) < 32 mmHg; (3) body temperature > 38°C or < 36°C; or (4) leukocyte count > 12 × 10^9^ cells/l or < 4 × 10^9^ cells/l.

Although the exact mechanisms of SIRS are unclear, in general, many scholars believe that SIRS is a clinical process that is characterised by generalised inflammatory hyper-reactivity caused by various severe clinical insults triggered by infectious factors and non-infectious host stimulatory agents. Extensive capillary leakage and mesenchymal oedema caused by various inflammatory mediators and cytokines lead to reduced blood perfusion of vital organs, microcirculatory disturbances, shock, and organ function decline or failure, ultimately resulting in multiple organ dysfunction syndrome (MODS) and multiple organ failure (MOF). When SIRS progresses to MODS and MOF, the mortality rate increases to a range of 30–80%, depending on the number of failed organs.^2^

Cardiovascular complications of SIRS include shock, pericardial effusion, and even non-specific ST–T changes in the electrocardiogram (ECG) that mimic acute myocardial infarction. However, ST-elevation myocardial infarction (STEMI) complicated by SIRS has rarely been reported, and most of the reported cases could not be classified as true myocardial infarction, according to cardiac enzyme levels and echocardiographic or angiographic findings.

A literature review performed with PubMed, using the keywords ‘systemic inflammatory response syndrome (SIRS)’ and ‘ST-elevation acute myocardial infarction (STEMI)’, identified few reports in the English language literature between 1990 and 2013. In contrast to our case, two published reports by Samimi-Fard *et al.*^3^ and van Diepen *et al.*^4^ found that patients with STEMI may present with SIRS after primary percutaneous coronary intervention (PPCI). Conversely, our case showed that SIRS may cause STEMI, which is an extraordinary finding, given the absence of relevant coronary stenosis.

Here, we report a case of SIRS-related myocardial infarction involving a 45-year-old man who developed SIRS secondary to diabetes-induced infection, followed by ST-elevation myocardial infarction with acute heart failure, fulminant diabetes, acute liver dysfunction, acute kidney dysfunction and rhabdomyolysis. Awareness of this type of myocardial infarction is critical to enable prompt diagnosis and treatment in similar cases.

## Case report

A 45-year-old man with a history of diabetes was admitted with cough, polyuria and polydipsia of one week’s duration. The patient had no medical history of hypertension and myocardial infarction. Upon physical examination, the patient was found to have a body temperature of 38.2°C, a respiratory rate of 23 breaths/min, a pulse rate of 106 beats/min and a systolic/diastolic blood pressure of 112/82 mmHg. A few moist crackles were apparent in the patient’s lungs.

Laboratory studies revealed a white blood cell count of 23.02 × 10^9^ cells/l with 88.6% neutrophils, and the following blood levels: alanine aminotransferase (ALT), 233 U/l (range 0–40); creatinine (Cr), 243.1 μmol/l (range 44–133); creatine kinase (CK), 36 762 U/l (range 26–174); plasma glucose, 49.1 mmol/l; fasting C-peptide, 0.01 ng/ml (range 0.7–1.9); two-hour postprandial C-peptide, 0.15 ng/ml; glycosylated haemoglobin (HbA_1c_), 8.4%; and C-reactive protein (CRP), 33.7 mg/l (range 0–5). The patient’s creatine kinase-MB (CK-MB) concentration was > 500 ng/ml, and his troponin I concentration was > 180 ng/ml. Arterial blood gas analysis results showed the following: pH, 7.299; PO_2_, 16.80 kPa; PCO_2_, 4.01 kPa; base excess (BE), –10.8 mmol/l.

**Figure 1.**
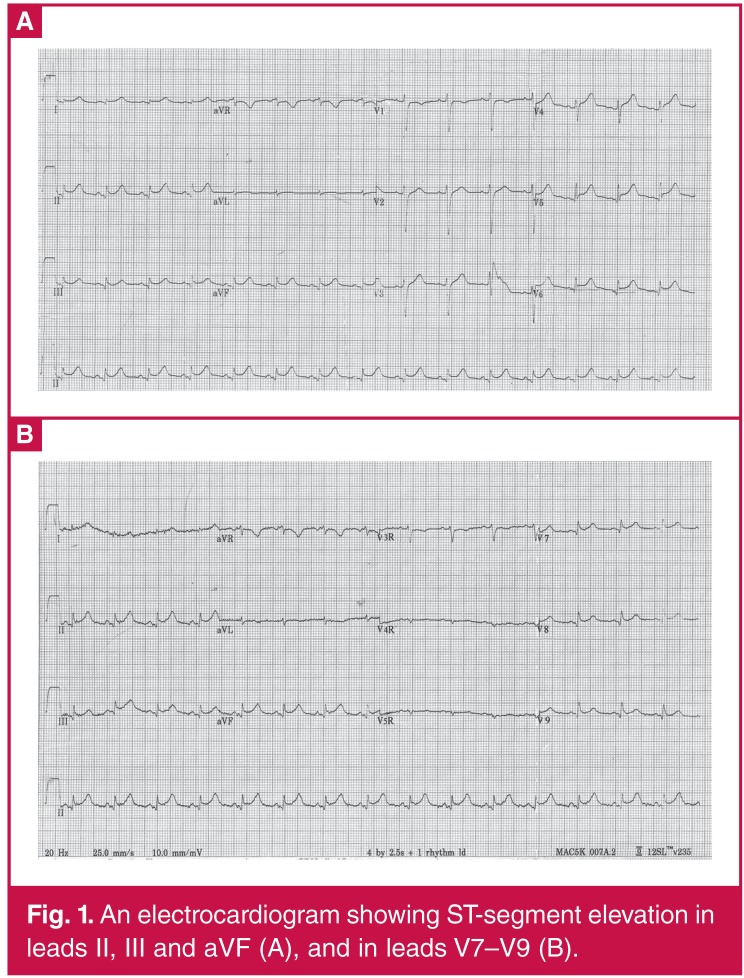
An electrocardiogram showing ST-segment elevation in leads II, III and aVF (A), and in leads V7–V9 (B).

**Figure 2.**
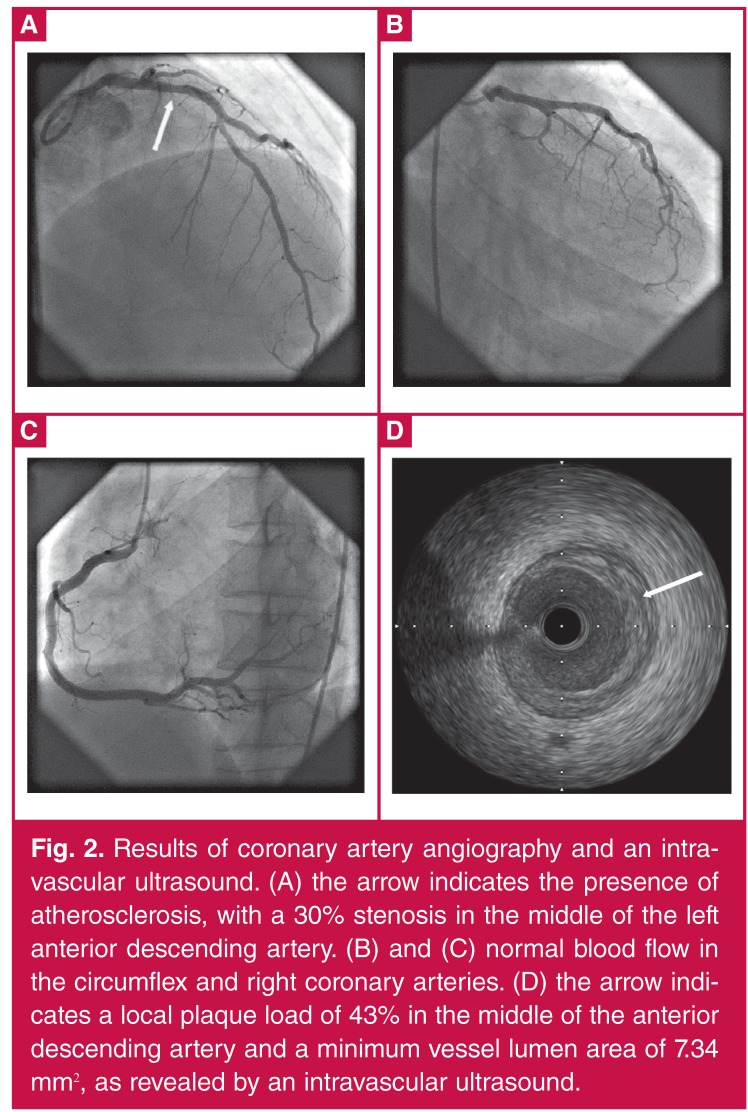
Results of coronary artery angiography and an intravascular ultrasound. (A) the arrow indicates the presence of atherosclerosis, with a 30% stenosis in the middle of the left anterior descending artery. (B) and (C) normal blood flow in the circumflex and right coronary arteries. (D) the arrow indicates a local plaque load of 43% in the middle of the anterior descending artery and a minimum vessel lumen area of 7.34 mm^2^, as revealed by an intravascular ultrasound.

A chest X-ray revealed a pneumonia infection of the lower right lung. An ECG showed ST-segment elevation in leads II, III, aVF and V7–V9 (Fig. 1A, B). Echocardiography revealed decreased left ventricular systolic function (ejection fraction: 32%) with left ventricular inferior and posterior wall motion abnormalities. Coronary angiography indicated no luminal narrowing in the left main coronary artery, circumflex or right coronary arteries, although atherosclerosis was apparent in the left anterior descending artery, with stenosis of 30% (Fig. 2A-C). Intravascular ultrasound results showed a local plaque load of 43% in the middle of the anterior descending artery, with a minimum vessel lumen area of 7.34 mm2 (Fig. 2D). Thus, myocardial infarction induced by coronary atherosclerosis and plaque rupture was excluded.

From the above findings, the patient was diagnosed with SIRS and MODS, which included acute inferior and posterior wall myocardial infarction with acute heart failure, fulminant diabetes, acute liver dysfunction, acute kidney dysfunction and rhabdomyolysis. The patient received a series of powerful treatments, including insulin intravenous infusion, antiplatelet therapy and anticoagulation therapy with aspirin, clopidogrel and low-molecular-weight heparin, diuretics, urine alkalisation, and anti-inflammatory antibiotic therapy.

The patient’s condition improved after treatment for one week; each organ regained normal function and the patient was discharged in a good clinical state after a total hospitalisation of 14 days. The follow-up visit in out-patient service one month after discharge showed that the patient’s blood test, ECG and echocardiography results were all normal.

## Discussion

Studies have shown that SIRS may produce stress hyperglycaemia, which has possible detrimental effects on the prognosis of patients.^5^ Elevated blood glucose levels may also predict mortality and length of intensive care unit (ICU) and hospital stay for trauma patients, and have been associated with infectious morbidity and prolonged need for mechanical ventilation.^6^,^7^ A strong link has been described between elevated blood glucose levels and the risk of critical illness in sepsis and SIRS.^8^ So, for this patient, whose blood glucose was reported out of range at 49.1 mmol/l, early active glycaemic control was extremely important. Two large randomised, controlled clinical trials have demonstrated that maintenance of normoglycaemia with intensive insulin therapy may reduce morbidity and mortality rates in critically ill patients.^9^,^10^

The case reported here represents true myocardial infarction, based on the changes in cardiac enzyme levels, as well as echocardiographic and ECG findings. To our knowledge, this is the first reported case of SIRS-induced myocardial infarction. Different from previous reports, there was no relevant coronary stenosis in this case, as shown by the coronary angiography and IVUS (Fig. 2).

A new clinical classification of myocardial infarction was proposed in the 2007 ESC/ACCF/AHA/WHF consensus conference,^11^ and clinically various types of myocardial infarction were classified by pathogenesis, as described in Table 1. In the present case, the myocardial infarction should be classified as type 2, and cardiac damage induced by SIRS is likely to be the most probable mechanism of acute myocardial infarction. Additionally, a hypodynamic circulation, especially one reflected in a notable reduction in the ejection fraction (32%) by echocardiography, as in this case, may also be related to myocardial infarction.

**Table 1:** Clinical classification of myocardial infarction (MI), as proposed at the 2007 ESC/ACCF/AHA/WHF consensus conference

*Type of MI*	*Description*
Type 1	Spontaneous MI related to ischaemia due to a primary coronary event such as plaque erosion and/or rupture, fissuring or dissection
Type 2	MI secondary to ischaemia due to either increased oxygen demand or decreased supply, e.g. coronary artery spasm, coronary embolism, anaemia, arrhythmias, hypertension or hypotension
Type 3	Sudden unexpected cardiac death
Type 4a	MI associated with primary percutaneous coronary intervention (PPCI)
Type 4b	MI associated with stent thrombosis as documented by angiography or at autopsy
Type 5	MI associated with coronary artery bypass grafting (CABG)

In recent years, many researchers have suggested that local and systemic inflammation may play an important role in the occurrence, development and complications of acute coronary syndrome (ACS).^12^ However, coronary angiography is essential to avoid the potentially lethal consequences of thrombolytic therapy in this type of myocardial infarction.

In summary, according to the results of glycated haemoglobin and the patient’s medical history, we may infer that the patient had a medical history of diabetes that was not well controlled. Pneumonia infection was induced by diabetes; therefore, we speculated that the patient developed SIRS secondary to diabetes-induced infection. As a result, a large number of inflammatory factors, including C-reactive protein, may have damaged the patient’s pancreas, heart, liver, kidney and skeletal muscle, leading to MODS.

## Conclusion

The present case demonstrates that SIRS may lead to multiple organ damage, and even to a clinical performance of coronary artery disease mimicking acute myocardial infarction. Urgent diagnosis by angiography is required. To enable prompt diagnosis and effective treatment in similar cases, clinicians should be aware of this type 2 myocardial infarction. Patients who smoothly obtain an inflammatory reaction peak under active treatment will likely experience good results.
